# Study of Geopolymers Obtained from Wheat Husk Native to Northern Mexico

**DOI:** 10.3390/ma16051803

**Published:** 2023-02-22

**Authors:** Claudia Alejandra Hernández-Escobar, Alain Salvador Conejo-Dávila, Alejandro Vega-Rios, Erasto Armando Zaragoza-Contreras, José Rurik Farias-Mancilla

**Affiliations:** 1 Centro de Investigación en Materiales Avanzados, SC, Miguel de Cervantes No. 120, Complejo Industrial Chihuahua, Chihuahua 31136, Mexico; 2 Instituto de Ingeniería y Tecnología, Universidad Autónoma de Ciudad Juárez—UACJ, Ciudad Juárez 32310, Mexico; 3 Centro de Innovación Aplicada en Tecnologías Competitivas, A.C. Calle Omega No. 201, Industrial Delta, León 37545, Mexico

**Keywords:** wheat husk, wheat husk ash, microwave curing, geopolymer

## Abstract

Agro-industrial wastes such as wheat husk (WH) are renewable sources of organic and inorganic substances, including cellulose, lignin, and aluminosilicates, which can be transformed into advanced materials with high added value. The use of geopolymers is a strategy to take advantage of the inorganic substances by obtaining inorganic polymers, which have been used as additives, e.g., for cement and refractory brick products or ceramic precursors. In this research, the WH native to northern Mexico was used as a source to produce wheat husk ash (WHA) following its calcination at 1050 °C. In addition, geopolymers were synthesized from the WHA by varying the concentrations of the alkaline activator (NaOH) from 16 M to 30 M, namely Geo 16M, Geo 20M, Geo 25M, and Geo 30M. At the same time, a commercial microwave radiation process was employed as the curing source. Furthermore, the geopolymers synthesized with 16 M and 30 M of NaOH were studied for their thermal conductivity as a function of temperature, in particular at 25, 35, 60, and 90 °C. The chemical composition of the WHA, determined by ICP, revealed a SiO_2_ content close to 81%, which is similar to rice husk. The geopolymers were characterized using various techniques to determine their structure, mechanical properties, and thermal conductivity. The findings showed that the synthesized geopolymers with 16M and 30M of NaOH had significant mechanical properties and thermal conductivity, respectively, compared to the other synthesized materials. Finally, the thermal conductivity regarding the temperature revealed that Geo 30M presented significant performance, especially at 60 °C.

## 1. Introduction

Crop production generates a substantial amount of waste, which has the potential to be transformed into value-added products. For example, wheat husk (WH) is a waste product obtained from wheat grain with an estimated content range of 15 to 20%. The wheat crop is the second largest produced worldwide [1]. The procedure for the removal of agricultural waste products (corn, rice, sorghum, sugar cane, and wheat) in Mexico involves open-air burning, which is a common practice. This method also allows the land to be cleared for later harvest, as well as controlling grasses, weeds, insects, and pests and facilitating the absorption of nutrients [2]. The calorific value determined for native wheat in Mexico exceeds 14.5 MJ/Kg [3].

Various strategies have been developed to take advantage of this natural resource. Blending WH with sustainable polymers has been explored [4]. Likewise, the current research on wheat husk ash (WHA) demonstrates that it can be used as a source to obtain biosiliceous substances, including silica aerogel, silica, silicon carbide, and silicon nitride, due to its high SiO_2_ content [1,5]. Another strategy is the geopolymerization, which is performed by mixing the solid precursor with an alkaline hydroxide solution such as sodium hydroxide (NaOH), potassium hydroxide (KOH), calcium hydroxide (Ca(OH)_2_), and an alkaline silicate or both [6]. In summary, the wastes from crop production are attractive alternatives for developing materials, especially geopolymers, due to the inorganic components that can be isolated from fly ash and obtained as by-products of certain calcination processes with the use of heat energy [7].

Additionally, there are few research reports on geopolymers from WHA and their thermal conductivity at different temperatures [8]. The reported work has generally focused on using wheat stem ash as a concrete reinforcement. However, both sources, although they are from the same plant, contain different silica contents [8]. The limitation to employing fly ash as a precursor of geopolymers is the aluminum content, which is indispensable to developing materials with high mechanical properties [9]. Nevertheless, the conventional methods for obtaining alumino-silicates are environmentally aggressive and expensive [10,11]. To solve this problem, many authors have added aluminum from other sources [12]. Nonetheless, the cost increase is a limitation of employing geopolymers as a commodity.

The methodological versatility of the geopolymerization process in adapting the geopolymers’ properties, depending on the material uses, is notable. Although geopolymers pose numerous production challenges relating to the curing time, temperature, type of precursor, and alkaline solution, they have high chemical resistance and excellent mechanical and thermal properties [13]. Strategies to accelerate the curing time involving heating-assisted [14], steam [15], and microwave-assisted [16] methodologies have been studied. Specifically, for microwave radiation, Chindaprasirt et al. [16] reported that 3 min of exposure is equivalent to 24 h of curing at 65 °C. Consequently, the geopolymer research field is constantly expanding.

In this paper, we report a study on geopolymers synthesized from WH native to northern Mexico. The focus of the research was to study the thermal conductivity at 25 °C and the mechanical properties regarding alkaline activator (NaOH) concentrations ranging from 16 M to 30 M. In addition, the thermal conductivity at 25, 35, 60, and 90 °C, of the geopolymers synthesized with 16 M and 30 M NaOH were studied.

## 2. Materials and Methods

### 2.1. Materials

The wheat husk (WH) was obtained from a local producer from Chihuahua State, Mexico. Sodium hydroxide (NaOH, Sigma-Aldrich, 98%, St. Louis, MO, USA), sodium silicate solution (Na_2_SiO_3_, Golden Bell, technical grade 97%, Zapopan, Jalisco, Mexico), distilled water (J.T. Baker, Phillipsburg, NJ, USA), and all reagents were used as delivered.

### 2.2. Wheat Husk Calcination

**Figure 1:**
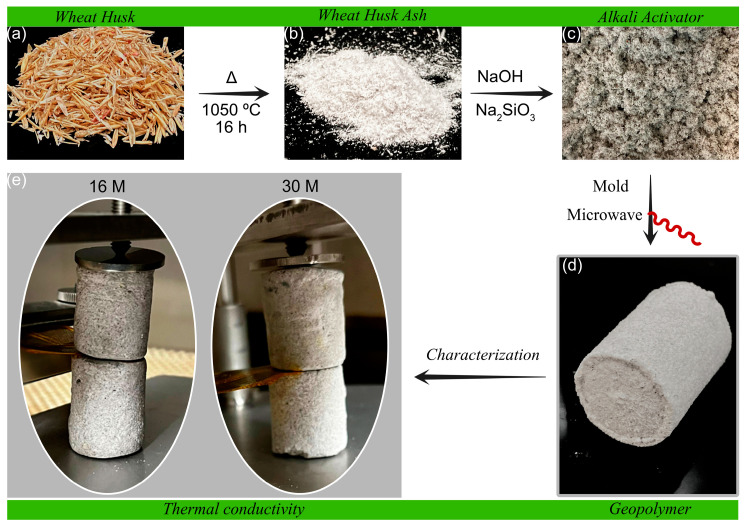
Flow diagram of the manufacturing process of geopolymers and their characterization: (**a**) wheat husk (WH); (**b**) wheat husk ash (WHA); (**c**) mixture of WHA with alkaline activator; (**d**) geopolymer; (**e**) thermal conductivity characterization.

The WH (1000 g, see Figure 1a) was heated at 100 °C for 8 h to dry the material. Then, it was ground in a mortar and calcined at 1050 °C for 16 h. The wheat husk ash (WHA) obtained here was a white powder (see Figure 1b).

### 2.3. Preparation of Alkali Solution

To prepare the NaOH solution, initially NaOH flakes were dissolved in the distilled water based on the required molarity. The concentrations of NaOH used in this investigation were 16 M, 20 M, 25 M, and 30 M. The solutions were prepared 24 h before synthesizing the geopolymers.

### 2.4. Geopolymerization

The geopolymerization was performed in 400 mL polypropylene beakers, mixing 35.7 g of WHA, 3.57 g of sodium hydroxide solution (16 M to 30 M), and 10.67 g of sodium silicate (see Figure 1c). The substances were mechanically stirred at 800 rpm for 3 min. The addition of sodium silicate and alkaline activator was to accelerate the geopolymerization. Then, the mixture was transferred into PVC molds previously lubricated with silicone and compressed in cylindrical form using a hydraulic compression molding press (Carver Inc., Wabash, IN, USA). Finally, the unmolded geopolymers were treated using a domestic microwave (DAEWO KOR-6LYB) for 3 min at 540 W (see Figure 1d). The products were named with the acronym Geo and the corresponding amount of alkaline activator, e.g., Geo 16 M (see Table 1).

### 2.5. Characterization

The functional groups were analyzed using a Fourier-transform infrared spectrometer (FTIR) (GX-FTIR, Perkin Elmer, Waltham, MA, USA) with an ATR accessory. The spectra represent the averages of 30 scans, with a resolution of 40 cm^–1^ and a spectral window ranging from 500 to 4000 cm^−1^. The inorganic elements were analyzed in WH and WHA samples using an inductively coupled plasma (ICP) mass spectrometer (Thermo, Scientific, iCAP 7400, Rochester, NY, USA). The thermal stability was studied using a thermogravimetric analyzer (SDT Q600, TA Instruments, New Castle, DE, USA) from room temperature to 800 °C in an air atmosphere and at a heating rate of 10 °C min^–1^. An X-ray diffractometer (X’Pert PRO RX04, Malvern Panalytical, Almeo Overijssel, The Netherlands) was used to determine the geopolymers´ crystalline structure. A scan range from 5° to 60°, a step size of 0.03330, and 60 s as the counting time were the analysis conditions. An electric hydraulic press (ELVEC, Mexico City, Mexico) with a capacity of 120 tons was used to evaluate the compressive strength. In order to test the specimens, the press was equipped with a CT-715H pump. The equipment resolution was 10 kg, with an accuracy rate of ±1 kg and a displacement of 25 mm. The BET specific surface area and pore size distribution of the geopolymers were determined by employing the Quatachrome NovaWin series volumetric gas adsorption instrument. The gas adsorption system uses nitrogen as the adsorptive component. Experiments for adsorption isotherms were performed at 77.35 K with a P/P_0_ relative pressure ratio of 0.050/0.100 (adsorption/desorption). Using the transient plane source technique, the thermal conductivity test was performed on a constant thermal analyzer (Hot Disk TPS 500, Västra Götaland, Sweden). The cylindrical-shaped samples had measurements of D = 2 cm and L = 3 cm. The assays were run at 25, 35, 60, and 90 °C in triplicate. Temperature control (for conductivity measurements) was performed inside an environmental chamber (Thermotron, SM 3.5, Holland, MI, USA). The sample was conditioned for 1 h prior to the analysis. The microstructure was analyzed utilizing a scanning electron microscope (SU3500, Hitachi, Chiyoda, Tokyo, Japan) operating at 10 kV and 10 mm working distances. The surface micrographs by SEM were taken using secondary electron detectors.

## 3. Results and Discussion

### 3.1. Functional Group Analysis

**Figure 2:**
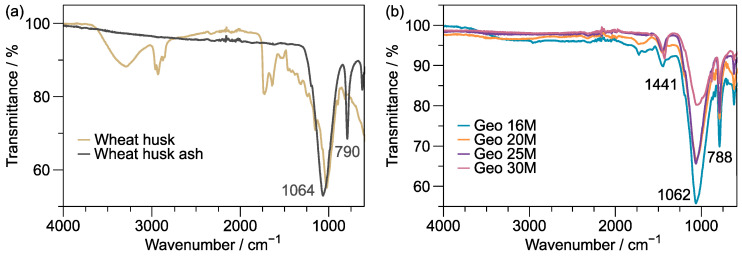
FT-IR spectra of the wheat husk (WH), WHA, and formulations of Geo 16M to Geo 30M: (**a**) WH, WHA; (**b**) Geo 16M, Geo 20M, Geo 25M, and Geo 30M.

Wheat husk (WH) waste contains organic and inorganic components, which may be different due to the type of source and place of harvesting [17]. It is well known that organic substances mainly comprise cellulose, hemicelluloses, and lignin [18]. Characterization tests by FT-IR can confirm the molecular vibrations of the specific functional groups of these substances (see Figure 2a). For example, the stretching vibration of the —OH groups appear at 3286 cm^–1^, and the absorptions at 2962 and 2922 cm^–1^ are assigned to the symmetric and asymmetric stretching vibration of the CH_2_ groups, respectively. The vibration of C=O from hemicelluloses is displayed at 1724 cm^–1^, while the C—O stretching vibration corresponding to the glucose rings occurs at 1024 cm^–1^. Hence, most molecular vibrations corresponding to polysaccharides are present [19].

Once calcinated, the wheat husk ash (WHA) is obtained. Compared with the WH spectrum, all organic compounds are degraded; therefore, only inorganic remain. The absorption at 1064 cm^–1^ was attributed to the asymmetric stretching of Si–O–Si, and the peak at 790 cm^–1^ corresponded to the symmetric stretching of Si–O–Si [20,21].

On the other hand, the characteristic bands of geopolymers appear similarly in the vibration regions of WHA (see Figure 2b). However, the peak at 1441 cm^–1^ is ascribed to carbonate type (O—C—O) groups due to the absorption of CO_2_ from the atmosphere, which is a difference when compared with the WHA spectrum [22,23].

The chemical compositions of WHA and WH are displayed in Table 2. The WHA contains SiO_2_, CaO, Al_2_O_3_, Fe_2_O_3_, K_2_O, MgO, Na_2_O, and P_2_O_5_. These substances are similar to those in other reported studies; however, they have different amounts. In this research, the SiO_2_ content in the WH was 81%, while the WH (native to Turkey) reported by Terzioglu et al. was 51% [24]. Zhang et al. also reported a SiO_2_ content of 54% in WH [8]. The percentage composition of SiO_2_ for rice husk ash is greater than 87% [8,25].

### 3.2. Thermal Stability

**Figure 3:**
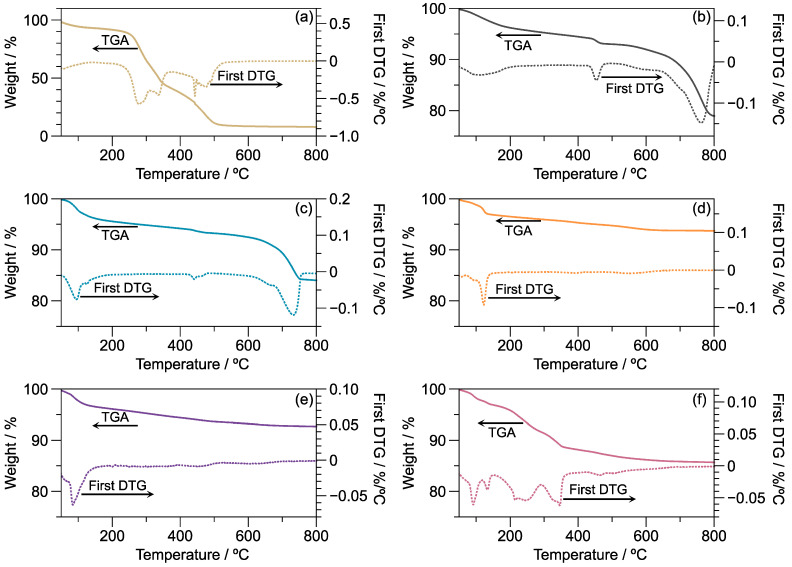
TGA and DTGA thermograms of (**a**) WH, (**b**) WHA, (**c**) Geo 16M, (**d**) Geo 20M, (**e**) Geo 25M, and (**f**) Geo 30M.

Figure 3 displays TGA traces and their first derivative (DTG). WH presents three degradation stages; the first occurs at 66 °C with 7% weight loss, assigned to water or other volatiles. The second stage has a maximum decomposition temperature of 276 °C with 50% weight loss, corresponding to cellulose and hemicellulose degradation [26]. Finally, the third transition occurs at 475 °C with 34% weight loss, ascribed to the lignin content. The content of WHA is in the order of 9% of the weight, comprised mainly of SiO_2_. The ash content of rice husks, according to TGA, is 12.6% [27]. These results are similar to the thermal degradation of other natural fibers [28].

Compared with WH, the WHA displays 78% weight of inorganic oxides. The inorganic trace shows 4% weight loss at 113 °C due to the absorbed water. Additionally, weight losses of 3% and 15% appear at 454.7 and 760.5 °C, respectively, which are assigned, in turn, to dihydroxylation and decarboxylation reactions [29].

The geopolymer formulations present a similar thermal behavior, showing a three-step degradation process. The first stage occurs close to 100 °C, representing 4% of the weight, which as for WHA is assigned to absorbed water. The second transition arises between 340 and 460 °C, representing from 1 to 4% of the weight of the sample, which is assigned to the dihydroxylation process [30]. The main difference between the geopolymers is observed in the last stage. Geo 16M presents this stage at 732.2 °C with 9% weight loss, while for Geo 20M to Geo 30M, this stage is displayed at around 520 °C, with weight losses ranging between 2 and 3%. This stage is assigned to the decarboxylation process. The variations in decomposition temperature in this region, according to the literature, depend on the role and interactions that carbonates present in the crystalline structure [31].

### 3.3. Crystal Structure

**Figure 4:**
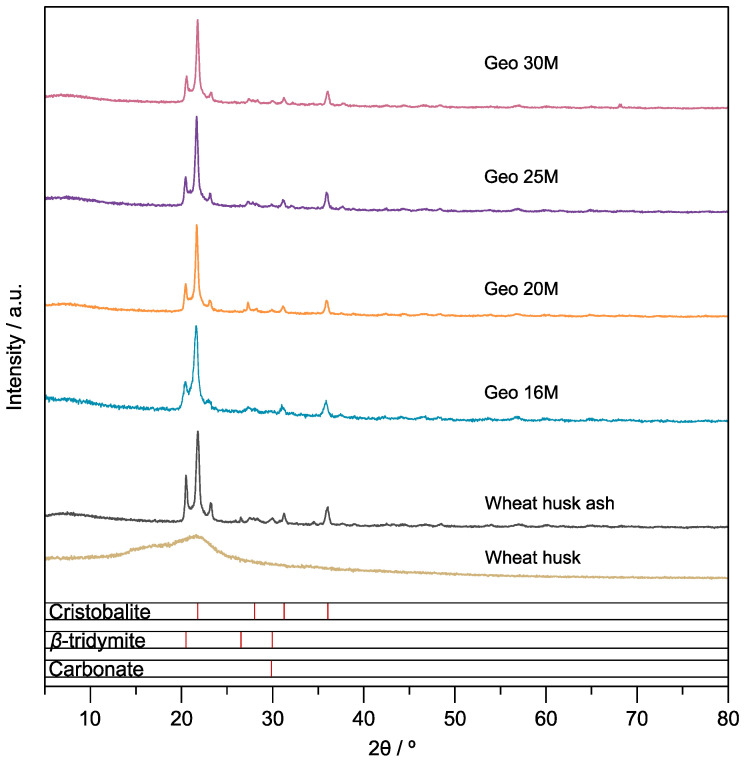
Diffractograms of WH, WHA, Geo 16M, Geo 20M, Geo 25M, and Geo 30M.

The diffractogram of WH does not present a crystalline structure (see Figure 4). The diffractogram after calcination (1050 °C) presents the characteristic peaks of SiO_2_ cristobalite (21.8°, 31.3°, and 36.1°) [32] and *β*-tridymite (20.4°, 21.6°, 35.8°) [33]. Both are polymorphs of crystalline silica (SiO_2_), presenting differences in the packaging of the silica tetrahedron, where the structure of the tridymite is confined in two layers. On the contrary, the structure of the cristobalite is three-layered. In addition, the cristobalite phase is transformed to tridymite at temperatures above 900 °C [34]. The cristobalite/*β*-tridymite ratio is close to 1:1. A high tridymite content is correlated with the degree of geopolymerization because of the larger number of silanol groups [33]. On the other hand, the peak confirms the presence of carbonates at 29.2° [35]. Geo 16 M shows decomposition of the crystalline structure owing to the geopolymerization process; therefore, the peaks are diminished and wide. Nevertheless, the crystalline structure was recovered when the geopolymers were formulated with high concentrations of NaOH (Geo 20M–30M) and cured under microwave radiation. This result can be explained by the formation of the hydrogen bond that hydroxide(-OH) produces [36]. In addition, the observed increase in XRD could be attributed to recrystallization caused by microwave radiation. However, what is not yet understood is the role of microwave radiation in the synthesis or curing of geopolymers [37].

Additionally, the XRD pattern of WHA (obtained at 600 °C from wheat native to Turkey) reported by Terzioglu et al. demonstrates a completely amorphous structure [38]. However, in another study by Terzioglu et al., it was reported that the formation of the cristobalite structure is the most significant crystalline phase during calcination at 1000 °C [24].

### 3.4. BET Specific Surface Area and Pore Size Distribution Analysis

The most popular method for determining the surface area of the materials is gas sorption (both adsorption and desorption) of dry solid powders. According to IUPAC, the porous materials are classified into microporous (D < 2 nm), mesoporous (2 nm ≥ D < 50 nm), and macroporous (D > 50 nm) [39]. Under this technique, the Geo 16M and Geo 30M formulations were studied (see Table 3). In addition, the pore size distributions of Geo 16M and Geo 30M were determined. Therefore, Geo 16M and Geo 30M were classified as mesoporous. The possible applications for these materials range from drug delivery [40,41] to catalysis [42], energy storage [43], and the removal of methylene blue [44], among others. Barbosa et al. [45] synthesized a mesoporous geopolymer from metakaolin and rice husk ash with potential application as an adsorbent to remove organic dye substances. In addition, the alkaline activator (KOH) concentration was close to 10 M and the specific surface area of the rice husk ash was 30 m^2^ g^–1^. This is an important issue for future research.

Moreover, Table 3 displays the specific surface areas of Geo 16M and Geo 30M. The WHA has been reported to decrease the specific surface area in correlation with the calcination temperature [24]. For example, a comparison of a specific surface area at temperatures of 600 and 1000 gave values of 7.21 m^2^/g and 0.11 m^2^/g, respectively [24]. In this study, the calcination of the WH was carried out at 1050 °C, and subsequently the geopolymer was obtained, so the result was similar to the reported value. According to Terzioglu et al. [24], the decrease in specific surface area can be explained by the collapse in the structural skeleton due to complete carbon consumption at higher temperatures or by the formation of glass-like structures at higher temperatures.

A relatively high volume of voids or pores is one of the characteristics required for geopolymers, especially in heavy metal adsorption [46]. The pore diameters range from nanometers to millimeters, with a total pore volume range of 30 to 90%. During the manufacture of the geopolymer, substances that promote gas generation or inclusion in the slurry mixing stage are added [47].

### 3.5. Mechanical Properties

The study of the mechanical properties was developed to understand other possible applications. The main application of geopolymers is in the construction industry. In addition, the results of the synthesized geopolymers were compared (without additives) with the values reported in the literature. The curing time using microwaves was based on the methodology reported by Chindaprasirt et al. [16]. These authors reported that microwave radiation enhanced the geopolymerization and compression strength based on findings from Chindaprasirt et al. [16], although the optimal conditions they reported combined microwave radiation and conventional heat curing, reducing the curing time and energy. Other variables have been studied, including the curing time (irradiation time) and potency. The results show a decrease in compressive strength for increasing power and also an increase in compressive strength related to the curing time [37].

**Figure 5:**
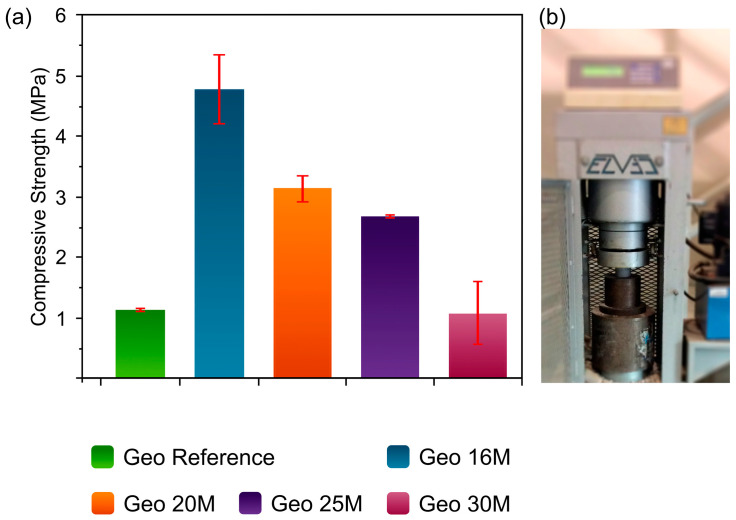
(**a**) Compressive strength values of the Geo Reference, Geo 16M, Geo 20M, Geo 25M, and Geo 30M samples. (**b**) Evaluation of the compressive strength of a sample.

Figure 5 illustrates the compressive strengths of the Geo 16M, Geo 20M, Geo 25M, and Geo 30M samples. As noted, Geo 16M shows a significant value close to 5 MPa of compressive strength, 5 times that of Geo 30 M. The diminished mechanical properties can be explained by the crystallinity of the geopolymer, since Geo 16M has the lowest crystallinity according to the XRD results. On the other hand, under these synthesis conditions, the microwave radiation has no significant effect on the compressive strength. The molarity of the NaOH increases in the formulations; however, the compressive strength decreases even though these are irradiated at the same power and time.

The geopolymer derivatives from natural resources generally have low mechanical properties; therefore, the addition of additives is required [12]. The compressive strength of WHA is similar to other geopolymer derivates from natural resources. For instance, Nadeem et al. reported on a natural soil-based geopolymer with compressive strength values ranging from 2 to 12 MPa [48]. Likewise, Hassan et al. reported one coconut-ash-based geopolymers with a compressive strength of 1.3 MPa [49].

### 3.6. Thermal Conductivity

Due to its porosity and low thermal conductivity, silica has been used as an insulating material [1]. Nevertheless, the structure can be modified by reactions such as geopolymerization, producing structures with closed pores and high thermal conductivity [50]. These two parameters depend largely on the viscosity, reaction system, and degree of geopolymerization, among others. Therefore, the differences in porosity and thermal conductivity between samples can be mainly attributed to the varying amounts of NaOH and silicate in the reaction system.

**Figure 6:**
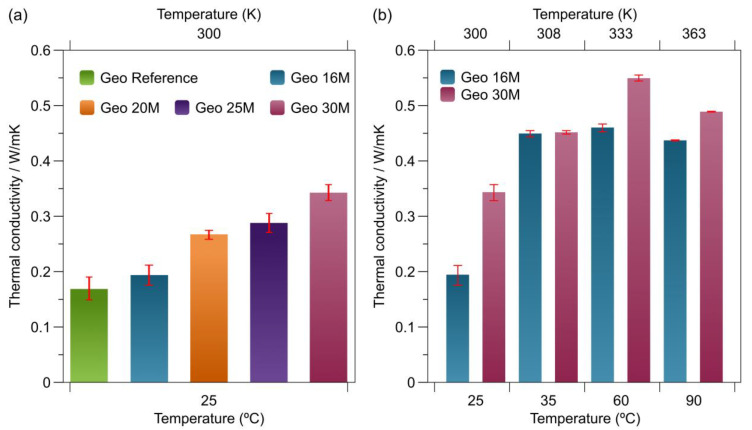
Bar plots of thermal conductivity: (**a**) Geo 16M, Geo 20 M, Geo 25M, and Geo 30M at 25 °C; (**b**) Geo 16M and Geo 30M at temperatures ranging from 25 to 90 °C.

Figure 6a illustrates the thermal conductivity of the Geo Reference, Geo 16M, Geo 20M, Geo 25M, and Geo 30M samples obtained at a temperature of 25 °C. These values are similar to other geopolymers coming from natural resources. For instance, Walbrück et al. reported on geopolymer foams reinforced with natural fibers, which presented thermal conductivity values ranging from 0.057 to 0.127 W/mK. This thermal behavior was attributed to the porosity of the samples [51]. As noted, the geopolymers formulated with higher concentrations of NaOH provide appropriate thermal conductivity and also possess a crystal structure (according to the XRD results). This finding can be explained by the formation of pores during the irradiation time, which is inversely proportional to the concentration of NaOH due to the water content.

Figure 6b displays a study of the thermal conductivity for the temperature of the Geo 16M and Geo 30M formulations. Regarding the formulation, Geo 16 M presents a value close to 0.45 W/mK from 35 to 90 °C. In addition, Geo 30M reaches a maximum value close to 0.55 W/mK at a temperature of 60 °C.

### 3.7. Morphology

**Figure 7:**
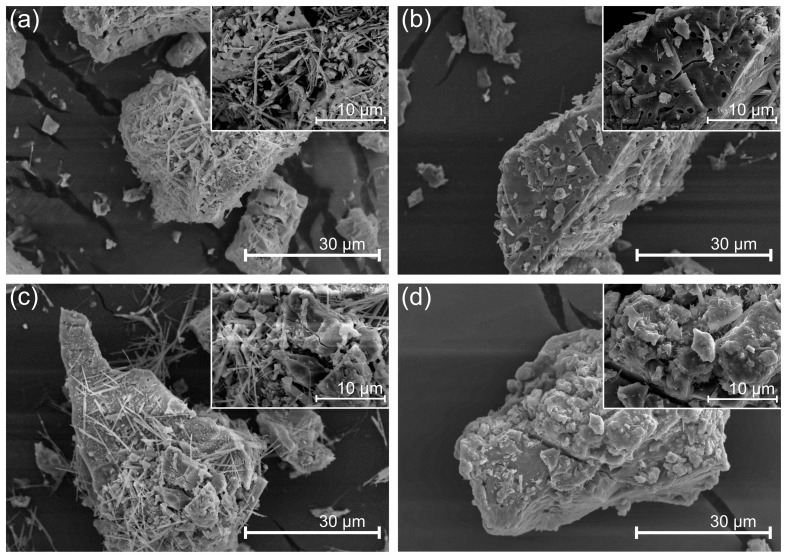
SEM micrographs of geopolymers: (**a**) Geo 16M; (**b**) Geo 20M; (**c**) Geo 25M; (**d**) Geo 30M.

A morphological analysis of the four geopolymer formulations was performed. Figure 7 reveals that the four systems present similar morphologies, showing needle-like crystalline structures. This characteristic agrees with the X-ray diffraction study, which indicated that higher alkaline concentrations increase the crystalline structure [52]. The main difference between the structures is the presence of higher porosity in the needle conglomerate framework in Geo 16M, followed by Geo 20M. The crystalline needles can be clearly observed in Geo 25M and Geo 30M; however, the structure appears to be more compact. It should also be noted that in the first two geopolymers, the surface appears to have a more significant presence of pores, while in the remaining two, the surface appears to be more solid. The porosity in these materials can be correlated with the low thermal conductivity [53], whereby it can be observed that Geo 16M acts as a better thermal insulator than the references. As such, it is understood that the alkaline activator in the highest concentrations produces a crystalline effect that favors thermal conductivity; however, it reduces its mechanical properties. The literature indicates that the alkaline activator produces amorphous geopolymers when enriched with aluminum [54].

## 4. Conclusions

In the present work, geopolymers (Geo 16M, Geo 20M, Geo 25M, and Geo 30M) from wheat husks native to northern Mexico, with a SiO_2_ content of 81%, were synthesized.

The high concentration of alkaline activator and curing by microwave radiation played a fundamental role in the crystal structure of the geopolymers, especially Geo 20M, Geo 25M, and Geo 30M, although a further study focused on the microwave radiation effect is suggested.

The compressive strength was higher when a NaOH concentration of 16M was used; however, the thermal conductivity was the lowest at this concentration. In addition, the compressive strength findings showed no significant effect when using microwave radiation during curing.

The study of the thermal conductivity regarding the temperature revealed that Geo 30M presents significant performance from 25 to 90 °C, especially at 60 °C.

Various applications can be explored from these results. For example, Geo 16M, because of its high mechanical properties and low thermal conductivity, is ideal for concrete coatings for the construction industry. On the contrary, Geo 30M has the potential to develop refractory bricks. In both cases, it is necessary to add additives to achieve their final application. Further research will focus on developing geopolymer formulations with balanced mechanical properties and thermal conductivity.

## Figures and Tables

**Table 1:** Geopolymer formulations consisting of wheat husk ash (WHA), sodium hydroxide (NaOH), and sodium silicate (Na_2_SiO_3_).

Sample	WHA (g)	NaOH [M]	Na_2_SiO_3_ (g)
Geo Reference	35.7	0	0
Geo 16M	35.7	16	10.67
Geo 20M	35.7	20	10.67
Geo 25M	35.7	25	10.67
Geo 30M	35.7	30	10.67

**Table 2:** Compositions of WH and WHA.

Sample	SiO_2_	Al_2_O_3_	Fe_2_O_3_	CaO	Na_2_O	MgO	K_2_O	P_2_O_5_
WHA	81.22	1.06	0.356	5.86	0.247	0.730	0.461	0.954
WH	N.D.	0.027	0.001	0.826	0.006	0.328	1.062	0.251

**Table 3:** BET characterization of Geo 16M and Geo 30M.

Sample	Average Nano Pore D (nm) BJH Method	*A_BET_* (m^2^/g) Multi-Point BET
Geo 16M	3.142	0.7723
Geo 30M	3.506	0.5999

## Data Availability

Not applicable.
